# Transcriptomic Analysis of Diethylstilbestrol in Daphnia Magna: Energy Metabolism and Growth Inhibition

**DOI:** 10.3390/toxics11020197

**Published:** 2023-02-20

**Authors:** Qi Li, Qian Zhao, Jiahua Guo, Xi Li, Jinxi Song

**Affiliations:** Shaanxi Key Laboratory of Earth Surface System and Environmental Carrying Capacity, College of Urban and Environmental Sciences, Northwest University, Xi’an 710127, China

**Keywords:** diethylstilbestrol (DES), *Daphnia magna*, chronic toxicity, transcriptome, energy metabolism

## Abstract

With the widespread use of diethylstilbestrol (DES), it has become a common contaminant in the aquatic environment. It is toxic to a wide range of aquatic organisms, disrupting the water flea growth and further interfering with several ecosystem services. Nevertheless, the molecular mechanism of DES in water fleas is still unexplicit. In this study, the 21-day chronic test showed that a negative effect of growth and reproduction can be observed with DES exposure. Subsequently applied transcriptomic analysis illustrated the molecular mechanism in mode freshwater invertebrate *Daphnia magna* (*D. magna*) exposed to 2, 200, and 1000 μg·L^−1^ of DES for 9 days. Meanwhile, exposure to DES at 200 and 1000 μg·L^−1^ significantly restrains the growth (body length) and reproduction (first spawning time) of *D. magna*. Identified differentially expressed genes (DEGs) are majorly enriched relative to energy metabolism, lipid metabolism, the digestive system, transport and catabolism pathways which were remarkably changed. These repressed and up-regulated pathways, in relation to energy synthesis and metabolism, may be the reasons for the reduced body length and delayed first spawning time. Taken together, this study revealed that DES is a threat to *D. magna* in the aquatic environment and clarifies the molecular mechanism of the toxicity.

## 1. Introduction

Freshwater crustacean *Daphnia magna* (*D. magna*) is the primary consumer of algae, bacteria, and protozoans, and is the primary forage for fish that delivers important ecological services in an aquatic ecosystem [1,2]. Because *D. magna* is susceptible to various toxic substances, the adverse effects on *D. magna* population and health probably disturb the multiple ecosystem services it provides [3]. In view of this, *D. magna* is frequently applied to hazard assessment and the classification of emerging chemicals, owing to its widespread distribution, short life cycle, sensitivity to chemical substances, parthenogenesis, and genetic homology [4,5,6]. With the application of *D. magna* in various chemical treatments, excellent ecotoxicological data have therefore been collected. For instance, most ecotoxicity data include the median effective concentration (EC_50_) and physiological indicators based on growth and reproduction endpoints. Yet, the molecular mechanism of chemicals in *D. magna* remains unknown, and concerns have been raised about the underlying mechanisms of adverse effects. Recent advances in ecotoxicology have shown that acute toxicity tests of chemicals have been transformed into chronic tests at environmentally relevant concentrations in order to elucidate the causes of these changes at the level of gene expression patterns [7]. Accompanied with the utilization of the quantification of total genome gene expression analysis, known as transcriptome analysis, it is possible to identify biological pathways disrupted by toxic substances [8,9]. This technique has been used to study the transient transcriptome alteration in *D. magna* after acute exposure to 17β-estradio to evaluate the dysregulated pathways [10]. Nevertheless, investigations related to the mechanism of action of diethylstilbestrol (DES) at the genetic level are still lacking.

DES is a typical synthetic estrogen, which is prescribed for preventing miscarriages, regulating estrogen secretion and animal growth [11,12]. Although DES have been prohibited for utilization in aquaculture and animal husbandry in many countries [13], it remains a threat to human health due to its illegal use in livestock production which leads to growing annual emissions [14,15]. The massive use has resulted in the content of DES in aquatic products at a μg/kg level; this accumulation might be a health threat to people and organisms [14]. Moreover, concentrations of DES as high as 24.9–102 ng·L^−1^ and 7.2–16.9 μg·L^−1^ were detected in some Chinese rivers and fisheries, respectively; this phenomenon showed that DES cannot be completely removed by a treatment plant but exists in natural water bodies for a long time [16,17]. Hence, DES existing in an aquatic environment may disrupt the health of organism. In *D. magna*, 17β-estradio interferes with RNA transport and signal transduction by competitively binding receptors, and affects the pathways related to steroid biosynthesis, lysosome, the intestinal immune system, and metabolism of active compounds [10]. Since DES has a stronger estrogenic activity than 17β-estradio, it can be inferred that DES treatment may cause similar genetic changes [18,19]. Laboratory studies demonstrated that exposure to DES caused reproductive effects in daphnids, and altered steroid metabolism capacities [20]. Successive DES treatments of F0 and F1 significantly inhibited the reproductive ability of offspring and molting frequency [21], but there was no research studying the chemical potential toxicity mechanism. At present, the application of transcriptome analysis has been a common method for identifying the toxic mechanism of the contaminant at a genome expression level [22,23]. While the chronic toxicological effects of DES on *D. magna* have been partially elucidated, the molecular mechanism of its inhibition of growth and reproduction based on the transcriptomic method remains poor.

In this study, the adverse effects of DES on the growth and reproduction of *D. magna* were evaluated on physiological and molecular levels by analyzing the alterations in physiological parameters and gene expression patterns. The chronic treatments were exposed to the series concentrations (20, 200, 1000 μg·L^−1^) of DES for 21 days to obtain physiological changes (body length, first spawning time, number of neonates, and molting frequency) of *D. magna*. Moreover, the molecular mechanisms of toxic effects were revealed by the changes in the transcriptome. Our hypothesis was that the exposure to range concentrations of DES may reduce body length, delay the reproduction time, and eventually result in growth and reproduction toxicity. In addition to the physiological changes, signaling pathways involved in energy metabolism, lipid metabolism, the digestive system, transport, and catabolism would be remarkably hindered. The objectives of this study were (1) to determine whether DES affects the life history parameters of *D. magna* at exposure concentrations; (2) to confirm the hypothesis by preliminarily clarifying the molecular mechanism of DES underlying the growth and reproduction inhibition.

## 2. Materials and Methods

### 2.1. D. magna Culture Maintenance

*D. magna* with homologous genes were provided by the Guangdong Provincial Laboratory Animal Research Institute. The *D. magna* were cultured in 2 L beakers containing artificial M4 medium with pH 7.8 ± 0.02, and the medium was renewed three times a week. All testing organisms were cultured in an incubator with the following conditions: a constant temperature of 21 ± 1 ℃, photoperiod of 16 h lightness:8 h darkness, and a light intensity of 1000–1500 lux. The *D. magna* were fed with green alga *chlorella* at a level of 2 × 10^6^ cells/mL (0.16 mg total organic carbon) daily per organism.

### 2.2. Chemicals

The DES (CAS No. 6998-97-1; purity ≥ 99%) and dimethylsulfoxide (DMSO; CAS No. 67-68-5; purity ≥ 98%) were purchased from Shanghai Yuanye Bio-Technology Co., Ltd. (Shanghai, China). A previous study showed that less than 0.1% (*v*/*v*) DMSO was not toxic to *D. magna* [24]. The DES stock solutions were made by dissolving in DMSO and were sonicated in a water bath for 30 min. All experiments have solvent control groups. Other chemicals used for preparing M4 medium were at least reagent grade.

### 2.3. Acute and Chornic Toxicity Test

Referring to the OECD 202 guideline for daphnid, acute immobilization test [25], acute toxicity tests was conducted to study the reference toxicant (potassium dichromate) and DES. Using the range-finding method to assess the EC_50_, the DES experimental concentration was determined to be 1, 1.5, 2, 2.4, 2.8, and 3 mg·L^−1^ (four replicates per concentration), and 10 mg·L^−1^ stock solution was prepared with M4 medium applied for an acute test. During the experiment, test neonates (<24 h) were not fed and per replicate consisted of five neonates. After exposure for 48 h, the immobilized neonates were counted to draw a concentration–response curve to then calculate the 48 h EC_50_.

Following the OECD 211 guideline [26], we performed the chronic toxicity test. Neonates were exposed to a solvent control group and three treatment groups with concentration of 20, 200, 1000 μg·L^−1^ for 21 days. Each neonate was cultured in a 50 mL glass beaker containing 20 mL test solution, and per group consisted of 20 replicates. The test solutions were renewed; meanwhile, the test organisms were fed every two days. During the 21 days of exposure, the time of first spawning, molting frequency, and number of neonates were recorded every day per organism. The newborn offspring were removed from the solutions after being counted daily. The body length, from the top of the head to the base of the caudal spine, of each test organism was measured by a stereomicroscope at 7, 14, and 21 days. In the experimental groups, all physiological alterations of organisms were recorded.

With reference to the above chronic test, a short-term experiment exposed for 9 days was conducted to analyze the alterations of the transcriptome. The experiment method was the same as the chronic test; the only difference was that each group consisted of 36 replicates to guarantee the biomass for sequencing. After test organisms completed their first spawning at day 9, daphnia in each treatment were randomly divided into three groups that each contained 12 individuals placed into 1.5 mL cryogenic tubes, snap frozen in liquid nitrogen. All samples were stored at −80 °C before total RNA isolation.

### 2.4. RNA Sequencing

Total RNA in daphnia samples were extracted and the concentration and purity of total RNA were measured by Nanodrop 2000 (Thermo Scientific, Wilmington, DE, USA). 2100 Bioanalyzer (Agilent, Santa Clara, CA, USA) was used to measure the concentration and purity of total RNA, and the RIN value of each sample was above 7.5. Total RNA samples were submitted to next-generation sequencing analysis (NGS) services with the application of Illumina HiSeq X Ten (Nanjing Personal Gene Technology Co., Ltd., Nanjing, China). The mapped reads of each sample varied from 52,595,459-57,663,891, with the coverage being from 94.78% to 95.77%.

### 2.5. RNA-Seq Data Analyses

For raw reads in the Fast Q format, the 3′ end _band adapter sequence and the low quality value (QV < 20) were deleted utilizing Cutadapt. The trimmed reads were mapped to the reference genome sequence of *D. magna* (GCA_003990815.1) using the HISAT2 (http://ccb.jhu.edu./software/hisat2/index.shtml (accessed on 16 November 2020)) software [27,28]. Following this, the read count value for each gene was estimated using the HTSeq statistics. The fragments per kilobase per million fragments (FPKM), serving as the gene expression level of read counts, were normalized to compare the expression levels. In order to verify the consistency of the three replications between groups, Pearson’s correlation analysis was estimated to examine the correlation of gene expression levels between samples. On the basis of gene expression, the similarities between solvent control and treatments were indicated by the utilization of principal component analysis (PCA) in the “DESeq” package of R software. The expression levels altered in DES-treated groups compared to solvent controls named differentially exposed genes (DEGs) were identified using the DESeq package, satisfying the cutoff: |log2 Fold Change (FC)| > 1 and adj *p* value < 0.05. The visual volcano plots and heat maps were generated, respectively, using the “ggplots2” and “Pheatmap” software packages. The Venn diagrams of different treatment groups were plotted to show the number of DEGs. The biological functions of DEGs were manifested by proceeding Gene Ontology (GO) and the Kyoto Encyclopedia of Genes and Genome (KEGG) pathways analysis.

With respect to GO enrichment, analyses were conducted with application of the “TopGO” package; the annotations of DEGs contained the molecular function (MF), biological process (BP), and cell component (CC). Significant enrichments of GO terms were deemed when the *p* value < 0.05. With regards to KEGG pathway analysis, the *p* < 0.05 was considered as a necessary condition to determine the enriched pathways.

### 2.6. Quantitative Real-Time PCR

In order to verify the gene expression profile obtained by RNA-seq, the expression levels of genes related to the digestive system and amino acid metabolism pathways were studied, and the following genes were measured for verification, including nieman C2 protein (*npc*2*)* and 4-aminobutyrate aminotransferase (*abat*), by real-time quantitative polymerase chain reaction (qRT-PCR). The expression levels of the above genes were subjected to normalization of a housekeeping gene, β-actin butler (*β-actin*). The special PCR process can be found in the Supplementary Data.

### 2.7. Statistical Analyses

In this paper, GraphPad Prism 8 software (San Diego, CA, USA) was applied to determine the statistical significance between treatments. The differences in physiological parameters between the DES treatment groups and solvent control groups were evaluated by one-way analysis of variance (ANOVA), followed by Dunnett’s post hoc test and *p* < 0.05 was regarded as statistically significant. To determine the correlation between NGS and qRT-PCR, Pearson correlation analysis was adopted, and the significant correlation was limited with *p* < 0.05.

## 3. Results

### 3.1. Effects of Acute and Chronic Toxicity Test of DES

**Figure 1:**
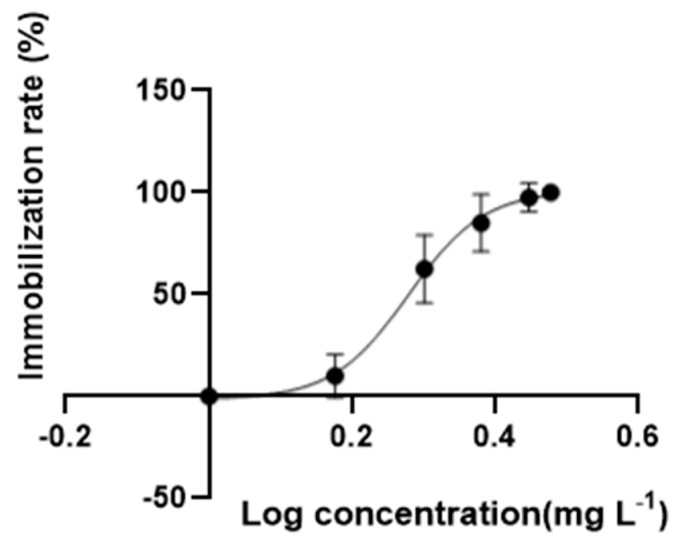
The immobilization rate of DES during 48 h test. Data are mean ± SD (*n* = 8).

**Figure 2:**
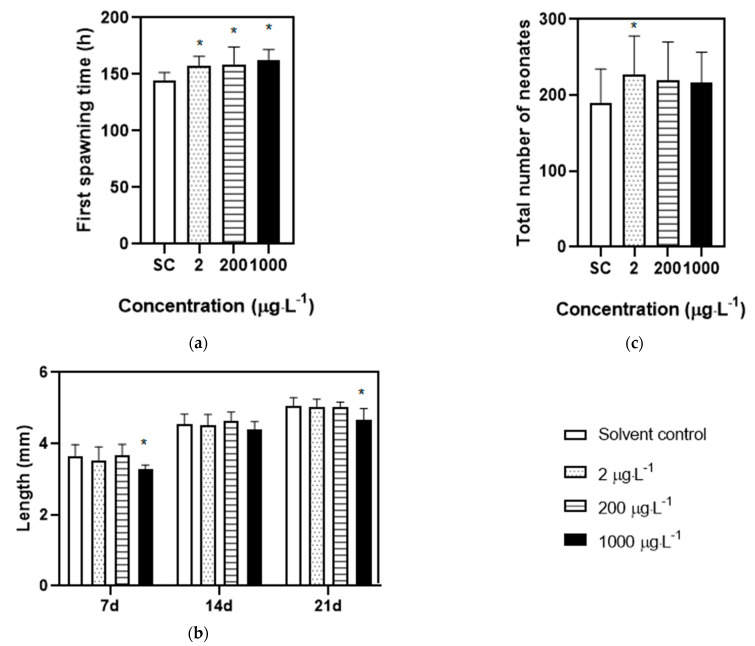
Physiological parameters of *D. magna* exposed to DES during chronic test for 21 d. (**a**) The first spawning time of *D. magna*; (**b**) the body length of *D. magna* at 7, 14, 21 d; (**c**) the total number of neonates of *D. magna*. “*” suggesting the parameters are statistically different between treatment groups and solvent control groups (*p* < 0.05 and *n* = 20).

In an acute exposure test, no immobile behavior was observed in solvent control groups, while the swimming behavior of daphnia treated with DES were inhibited after exposure for 48 h. The concentration–response curve for the immobilization of DES was present in Figure 1; the EC_50_ of 1.89 mg·L^−1^ with a 95% confidence interval range from 1.81 to 1.91 mg·L^−1^ was assessed for *D. magna*. Based on the above EC_50_, the range of concentrations for the chronic test and transcriptome analysis were determined. In the chronic toxicity test, no immobile organisms were observed in all treatments. Exposure to the low (2 μg/L), medium (200 μg/L), and high (1000 μg/L) concentrations of DES remarkably delayed the first spawning time relative to solvent control groups (Figure 2a). Moreover, the DES treatment at the highest level significantly reduced the body length of *D. magna* compared with solvent control groups at 7, and 21 days, meaning that the growth of the organisms was inhibited during development (Figure 2b). In the medium to highest level of DES treatments, a tendency to decrease the number of neonates was observed but with no significance (Figure 2c). Conversely, an exposure to low concentrations of DES significantly increased the total number of neonates (Figure 2c). In addition, no significance was observed in all treatments of molting frequency (Figure S1).

### 3.2. Transcriptome Analysis

#### 3.2.1. Differentially Expressed Genes

**Figure 3:**
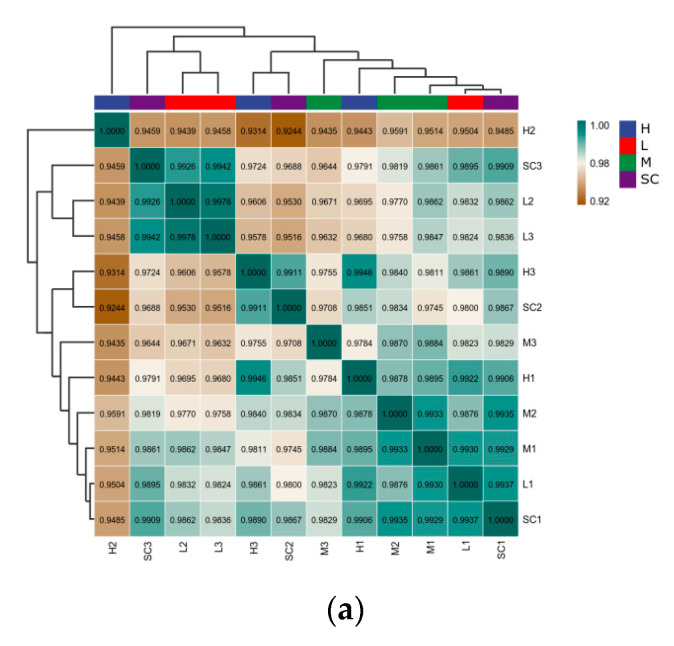

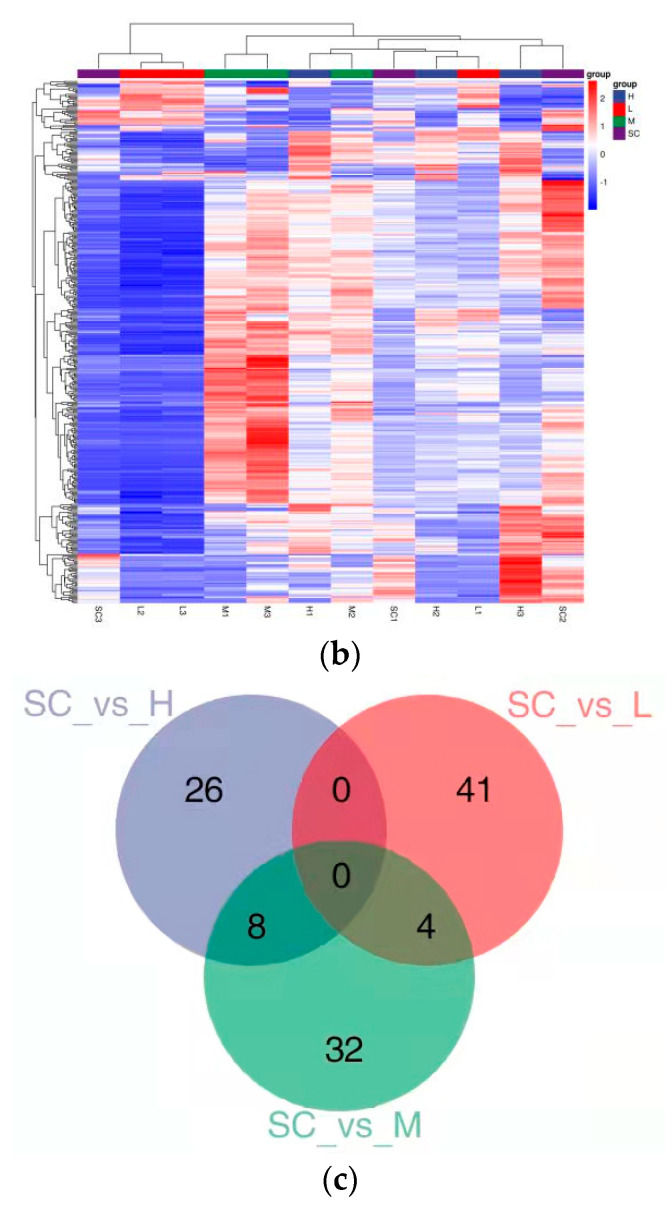
Transcriptomic profiles of *D. magna* after 9 d exposure to diethylstilbestrol (DES). (**a**) Correlation analysis of patterns of gene expression in solvent control groups and DES treatment groups; (**b**) a heatmap of centered and scaled FPKM value of DEGs in solvent control groups and DES treatment groups; (**c**) Venn diagram of the number of DEGs in each DES treatment group. SC: solvent control; L: low; M: medium; H: high.

A total of 13,137 transcripts were examined in *D. magna* in all treatments. With regard to gene expression patterns, a correlation coefficient no less than 0.93 was detected among the replications in each treatment, demonstrating a high co-correlation between samples (Figure 3a). In the PCA analysis (Figure S2), gene expression showed a resemblance to low treatments and solvent control treatments, while the medium and high treatment differed from the solvent control treatment, indicating that DES at medium to high levels may result in hazardous effects that are consistent with the results in heatmaps (Figure 3b).

With exposure to DES, at three treatment levels, about 41 (2 up-regulated and 38 down-regulated), 31 (6 up-regulated and 25 down-regulated), and 26 (10 up-regulated and 16 down-regulated) genes were identified as DEGs in daphnia, respectively (Figure 3c and Figure S3–S5). To confirm these results, the selective DEGs were conducted with qRT-PCR. The FPKM value of selective DEGs was in accordance with the mRNA expression levels measured by qRT-PCR, showing that the mRNA expression profiles in *D. magna* supplied by transcriptomic sequencing were quantified with confidence. For instance, the FPKM values of *npc*2 and *abat* were remarkably in correlation with the mRNA expression levels measured by qRT-PCR with *p* < 0.0001 and R2 = 0.82, and *p* = 0.0002 and R2 = 0.78, respectively (Figure S6, Table S1).

#### 3.2.2. Analyses of Gene Ontology and Functional Pathway

GO analysis was conducted to study the main biological functions of DEGs in the CC, MF, and BP. The top 20 GO enrichment terms in the low, medium, and high DES treatment groups were summarized (Figures S7–S9). In short, the DEs detected at low concentrations were mainly related to lipid biosynthesis processes (e.g., GO: 0008610 lipid biosynthesis process, 0006631 fatty acid metabolism process). Some GO terms were associated with energy metabolism in the medium treatment groups (e.g., GO: 0006486 protein glycosylation, GO: 0016798 hydrolase activity). DEGs in the high treatment groups were primarily enriched in GO terms related to the biological processes of wax (GO: 0010025 wax biosynthesis, GO: 0010166 wax metabolism) and energy metabolism (GO: 0009058 biosynthesis, GO: 0071704 organic metabolism). Therefore, these biological processes played an important role in the inhibition of DES on the growth of *D. magna*. The results of GO analysis were proven by enriched pathways connected with cholesterol metabolism, lipid metabolism, cutin, suberine and wax biosynthesis, and lysosome; the details are shown in Table 1. Special pathways in conjunction with other foundational biological processes are shown in Table S2.

## 4. Discussion

The ecotoxicity of DES to *D. magna* raised attention to its residues in the environment. However, studies related to transcriptome analysis and the molecular mechanism of DES were still deficient. The present study showed that chronic exposure to DES adversely affected the growth and reproduction of *D. magna* and revealed the toxic mechanism on a genetic level. Since estrogen drugs were highly toxic to *D. magna*, they served as references to further clarify the molecular mechanism of DES. In this study, the hypothesis that DES treatments may interfere with the pathways associated with energy metabolism, lipid metabolism, digestive system, transport, and catabolism was confirmed by transcriptome analysis. Consistent with this hypothesis, the down-regulation of the genes involved in the digestive system was closely related to the delay of the first spawning time and the decrease in body length after short-term exposure to DES. Thus, the potential associations between enriched signaling pathways and phenotypic changes were discussed in the following sections.

### 4.1. Genes Related to Energy Metabolism

Nitrogen is an important component of amino acids, and plays an important role in the structure and metabolism of organisms [29]. In this study, nitrogen metabolism was significantly inhibited by DES at low concentrations, in which the expression level of the carbonic anhydrase (*ca*) gene was down-regulated. Carbonic anhydrase (*ca*) is an active metal enzyme ubiquitous in organisms; its active site contains zinc ions bound to hydroxide, which can capture and absorb carbon dioxide and then catalyze the hydration reaction of carbon dioxide to produce bicarbonate [30]. Bicarbonate and ammonia can produce a precursor of the arginine, which is named the ammonia formate acid ester; this complex reaction mainly includes three steps: phosphorylation of bicarbonate, formation of carbamate, and phosphorylation of carbamate [31]. It can be speculated that the inhibition of *ca* activity can lead to a decrease in the bicarbonate content and inhibition of arginine synthesis. In addition, it has been found that bicarbonate catalyzed by *ca* plays an important role in maintaining the balance of intracellular pH value [32]. Since arginine deficiency can reduce growth and protein deposition, the arginine supplement can promote the growth of fish [33]. In view of this, the phenomenon of delayed first reproduction time of *D. magna* at a low concentration may be related to the reduction in arginine content caused by *ca* down-regulation; the lack of vital proteins greatly weakens the process of energy metabolism.

Amino acids are the precursors of bioactive molecules such as neurotransmitters, second messengers, and cytokines, which control various cellular processes, and its metabolism disorders can lead to various pathological phenomena [34]. In this study, the up-regulated 4-aminobutyrate transaminase (*abat*) gene is involved in amino acid metabolism pathways under a high DES level. It has been documented that *abat* is mainly responsible for the conversion of the inhibitory neurotransmitter 4-aminobutyric acid into succinate hemaldehydes, and finally succinate [35]. Succinate is regarded as a danger signal to promote the inflammatory response in the immune system, with increased levels in chronic inflammatory and metabolic diseases [36]. Therefore, the increase in succinate level induced by up-regulated *abat* in vivo indicated that the *D. magna* might be involved in immune metabolism, which proved that DES had a toxic effect on the organism at a high concentration. In addition, as reported when the articular cartilage of mice was damaged, the expression of *abat* in articular chondrocytes was significantly up-regulated. The induction of *abat* expression not only leads to metabolic changes in chondrocytes, but also increases the content of succinic acid which can result in increased mitochondrial respiration and consumption of a lot of energy [37]. Studies have shown that the overexpression of *abat* can lead to the catabolism of chondrocytes, but inhibition of abat expression can maintain chondrocyte homeostasis. It can be inferred that the significant decrease in the body length of *D. magna* at the high concentration treatment may be attributed to the overexpression of *abat,* which reduces chondrocytes and might cause bone deformation.

### 4.2. Genes Related to Lipid Metabolism

Sterol is an important component of biofilms, which is ubiquitous in crustaceans and has various biological functions [38]. In the low concentration treatment in this study, the gene of methylsterol monooxygenase (*erg*25), involved in the steroid biosynthesis pathway, was significantly down-regulated, which was consistent with the phenotypic changes of *D. magna* with the delayed first reproduction time. In the process of sterol synthesis, the initial lanosterol undergoes three demethylation before the final product of lanosterol is formed. The first demethylation occurs directly on lanosterol, leading to the removal of the C-14 methyl group, and the remaining demethylation is catalyzed by *erg*25, which eventually transforms dimethyllanosterol into the precursor of ergosterol called yeast sterol [39]. The lack of *erg*25 can lead to the accumulation of the dimethyllanosterol intermediate and its metabolites in vivo [40], ultimately resulting in toxic effects on organisms. In addition, ergosterol plays an important role in maintaining intracellular sterol homeostasis, and its deficiency leads to reduced plasma membrane mobility and intracellular ATP levels [41]. In short, the toxicity of DES on *D. magna* at a low concentration may be related to the abnormal expression of the *erg*25 gene, due to the homeostasis disrupted by massive intermediates and metabolites accumulated in cells.

Wax esters are macromolecules composed of long-chain fatty alcohols esterified into fatty acids, which are low-density neutral lipids used to store energy and contribute to the buoyance of copepods [41]. In the medium and high concentration treatments, the expression of aliphaloyl-coA reductase (*far*) genes involved in the wax biosynthesis pathway was up-regulated. The production of wax esters consists of two catalytic steps: fatty acids are reduced to fatty alcohols by *far* at first, and then fatty alcohols are trans-esterified to fatty acids to generate wax esters [42]. With the combined actions of *far* and wax ester hydrolase, the contents of fatty alcohols and wax esters in organisms maintain the dynamic balance. The generation rate of wax esters is limited only by fatty alcohols, which has been confirmed [43]. Thus, it is speculated that the induction of *far* expression can promote the production of wax esters, but some studies have shown that wax esters in adult copepods can be transferred to eggs during reproduction to ensure the development of eggs [44]. Therefore, it was inferred that the wax esters in *D. magna* may be transferred to the eggs during the reproduction process to meet the energy demands of offspring development, resulting in the energy deficiency in parental organisms, which is further in line with the negative changes in body length and number of offspring in the first brood.

### 4.3. Genes Related to Digestive System

The biliary acid is formed from cholesterol in the liver which plays an important role in the digestion and absorption of dietary lipids [45]. In the medium and high concentration treatments, the gene expressions of Nieman C2 protein (*npc*2) and cholesterol ester hydrolase (*lipa*) involved in the cholesterol metabolism pathway were significantly changed. The major function of *lipa* is to hydrolyze cholesterol esters into cholesterol, and its induction contributes to a large accumulation of free cholesterol in the late lysosome and inhibits the lysosome [46]. *Npc*2 is a key transporter driven by electrochemical potential, which regulates the transfer of cholesterol from lysosomes to the endoplasmic reticulum at the late stage. The efficiency of transporting cholesterol to the endoplasmic reticulum by vesicle and non-vesicle pathways was reduced by the down-regulation of *npc*2 [47]. In addition, the distribution and content of cholesterol in cells being in a highly dynamic balance is an important guarantee to maintain the integrity of cell function [48]. Thus, DES exposure may disrupt the metabolic process of cholesterol in cells and even interfere with the cell function. Bile acid is an important hydroxylated steroid synthesized from cholesterol in the liver and used to facilitate the absorption of nutrients, such as lipids and fat-soluble vitamins in the gut [49]. It has been proven that normal cholesterol metabolism is necessary to maintain a balance of lipids and bile acids in liver cells [44]. Hence, it was speculated that the passive effects of growth and reproduction on *D. magna* might be closely related to the inhibition of *npc*2. The reduction of cholesterol in the endoplasmic reticulum would significantly affect the secretion of bile acid, leading to a decreased ability of *D. magna* to digest algae and absorb the fat soluble cellulose.

Protein is eventually digested into amino acids, which are essential for normal growth, development, repair, and energy absorption of organisms [50]. In this study, gene expression of trypsin (*prss*1_2_3) and collagen type I (*col*1*a*) involved in protein digestion and the absorption pathway were both up-regulated in the high concentration treatments. Pancreatic digestive enzymes play a central role in digestion and usually are transported to the small intestine to hydrolyze complex nutrients, such as the endopeptidase of *prss*1_2_3 which is capable of splitting polypeptides into oligopeptides and amino acids [50]. The up-regulation of the *prss*1_2_3 gene may have aggravated the energy deficiency due to increased energy consumption. *Col*1*a* is an extracellular matrix protein as well an important part of the tumor mesenchymal environment, which plays a vital role in disrupting cell adhesion and activating β1-integrin to promote the epithelial–mesenchymal transformation in pancreatic cancer [51]. The expression of *Col*1*a* in liver cancer cells was significantly up-regulated, meaning that its overexpression could have affected the integrity of tissues and could lead to the diffusion and metastasis of tumor cells [52]. In the present study, the decreased body length of *D. magna* observed with a high level of DES treatment may be connected with abnormal liver cells; diseased liver cells may lead to the intake of green algae still not being digested, which might aggravate the problem of energy deficiency.

### 4.4. Genes Related to Transport and Catabolism

In the present study, the expression of lysosomal acid lipase (*lipa*) and Niemannc1 protein (*npc*) genes involved in the transport and catabolism pathway were significantly changed at medium and high concentrations. *Lipa* is an important regulatory factor of lysosomal lipolysis; meanwhile, the hydrolysis of cholesterol esters and triglycerides in lysosomes under acidic conditions is closely related to the production of lipid mediators, macrophage M2 activation, and inflammation [53]. As it was proven that the dysfunction of the lysosome is connected with the abnormal expression of lysosomal autophagy genes, the lysosomal somatic autophagy could be effectively blocked by down-regulated *lipa* [54]. Thus, the overexpression of *lipa* may be related to the toxic effect of the lysosome in the *D. magna* response to DES, exhibiting inflammatory reactions and consuming massive energy, ultimately leading to impaired energy transport. Moreover, *npc* acts as a transport protein on the lysosome membrane, and down-regulation of the gene can cause a gradual accumulation of cholesterol triglycerides, non-metabolic substrates such as proteins, and can result in lysosomal storage disorders; the continuous and vast accumulation of undegraded substrates in the lysosome will eventually lead to cell dysfunction and even death [55]. Therefore, lysosomes may be hydrolyzed and apoptotic in response to DES stress. Sequentially, the normal energy transport was restrained, and we observed the phenomenon that the growth and reproduction of *D. magna* were inhibited. Hence, DES exposure may inhibit the transport and catabolism of energy, which can account for the adverse physiological effect.

## 5. Conclusions

Overall, chronic exposure to DES remarkably affects the growth and reproduction of *D. magna* at low, medium, and high treatment groups; whereas, no mortality was observed in all treatment groups. DES treatment resulted in a difference in the *D. magna* transcriptome, demonstrating that the down-regulation of pathways in relation to energy metabolism, lipid metabolism, digestive system, transport, and catabolism may limit food digestion, energy absorption, and metabolic processes, ultimately leading to reduced body length and delayed reproduction of *D. magna*. In summary, DES treatments can observably affect the physiological phenotypes and molecular pathways of *D. magna*. Applying a transcriptomic approach, the differentially expressed genes and disordered molecular pathways were identified. They are recommended to serve as biomarkers to fully elucidate their toxic mechanism to aquatic organisms and provide a warning of health risk.

## Figures and Tables

**Table 1:** KEGG pathways significantly enriched (*p* < 0.05) in *D. magna* exposed to DES.

Pathways	Category	Up-Genes	Down-Genes
**SC vs. L**			
Steroid biosynthesis	Lipid metabolism	-	*meso*1, *erg*25
Terpenoid backbone biosynthesis	Metabolism of terpenoids and polyketides	-	*pdss*1
Nitrogen metabolism	Energy metabolism	-	*ca*
Cell adhesion molecules (CAMs)	Signaling molecules and interaction	-	*cntnap*2
**SC vs. M**			
Cholesterol metabolism	Digestive system	-	*npc*2
Lysosome	Transport and catabolism	*hgsnat*	*npc*
Cutin, suberine, and wax biosynthesis	Lipid metabolism	*far*	-
Glycosaminoglycan degradation	Glycan biosynthesis and metabolism	*hgsnat*	-
**SC vs. H**			
Cholesterol metabolism	Digestive system	*lal*	*npc*1, *npc*2
Lysosome	Transport and catabolism	*lipa*	*npc*1, *npc*2
Cutin, suberine, and wax biosynthesis	Lipid metabolism	*far*	-
Alanine, aspartate, and glutamate metabolism	Amino acid metabolism	*abat*	-
Protein digestion and absorption	Digestive system	*prss*1_2_3, *col*1*a*	-

## Data Availability

The data presented in this study are available upon request from the corresponding author.

## Supplementary Materials

The following supporting information can be downloaded at: https://www.mdpi.com/article/10.3390/toxics11020197/s1, Figure S1: Effects of DES exposure to *D. magna* in molting frequency; Figure S2: Principal component analysis (PCA) of DEGs of *D. magna* exposed to DES for 9d in transcriptomic analysis; Figure S3: Gene expression profiles of *D. magna* after 9 d exposure to DES at 2 μg L^−1^ volcano plot compared with solvent control groups; Figure S4: Gene expression profiles of *D. magna* after 9 d exposure to DES at 200 μg L^−1^ volcano plot compared with solvent control groups; Figure S5: Gene expression profiles of *D. magna* after 9 d exposure to DES at 1000 μg L^−1^ volcano plot compared with solvent control groups; Figure S6: qRT-PCR and RNA-seq correlation analysis results diagram of selected genes. *NPC2:* Niemann-Pick C2 protein; *abat:* 4-aminobutyrate aminotransferase; Figure S7: After a 9-d of exposure to different concentrations of DES, the top 20 enrichment terms for each category were significantly higher in the 2 μg L^−1^ treatment group than in the solvent control; Figure S8: After a 9-d of exposure to different concentrations of DES, the top 20 enrichment terms for each category were significantly higher in the 200 μg L^−1^ treatment group than in the solvent control; Figure S9: After a 9-d of exposure to different concentrations of DES, the top 20 enrichment terms for each category were significantly higher in the 200 μg L^−1^ treatment group than in the solvent control; Table S1: Primers utilised for quantitative polymerase chain reaction (qPCR) validation of gene expression; Table S2: List of fundamental pathways affected by a 9-day exposure to DES.
