# Supplementary material for: Transcriptomic Analysis of Diethylstilbestrol in Daphnia Magna: Energy Metabolism and Growth Inhibition
Source: Toxics. 2023 Feb 20;11(2):197. doi: 10.3390/toxics11020197 (PMC9962875; doi:10.3390/toxics11020197)
Supplement: Supplementary file 1 [file toxics-11-00197-s001.zip › toxics-2179080-supplementary.pdf]

Quantitative Real-Time PCR Analysis

We performed relative quantification of gene expression by fluorescent quantitative PCR (TIB8600, Triplex International Biosciences, China). Initially, qualified and quantified total RNA was reverse transcribed into cDNA using the PrimeScript TM 1st stand cDNA Synthesis Kit. The gene-specific qRT-PCR primers for *D. magna* mRNA quantification are presented in Table S1. The PCR reaction system consisted of 10 μL of 2 × SYBR real-time PCR premixture, 0.4 μL of LPCR-specific primer F (10 μM), 0.4 μL of PCR-specific primer (10 μM), 1 μL of cDNA and 8.2 μL of RNase free dH_2O per 20 μL. The reaction procedure was conducted in 3 replicates under the following conditions: first thermal denaturation at 95 °C for 5 min, after which 40 cycles were performed following the steps 95 °C for 15 s, 60 °C for 30 s. In addition, melting curve analysis was performed on all samples at the end of every run.

Table S1. Primers utilised for quantitative polymerase chain reaction (qPCR) validation of gene expression.

Gene ID	Gene	Forward Primers	Reverse Primers
LOC116931413	*NPC2*	CGTGTCTGAGCCCATTACCA	CCAGATTGCCTTTGCGTTCA
LOC116927431	*abat*	ACACCTGGATGGGTGATCCT	GCTTCAAAAGGTTGTCGCGT

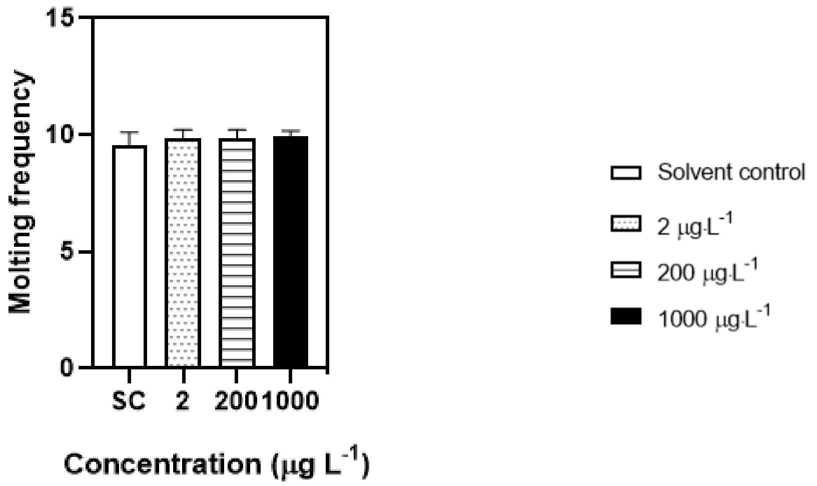

Figure S1. Effects of DES exposure to *D. magna* in molting frequency.($p < 0.05$; $n = 20$).

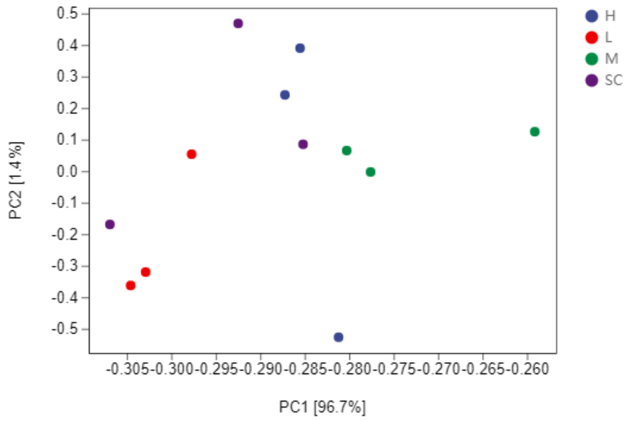

Figure S2. Principal component analysis (PCA) of DEGs of *D. magna* exposed to DES for 9d in transcriptomic analysis.

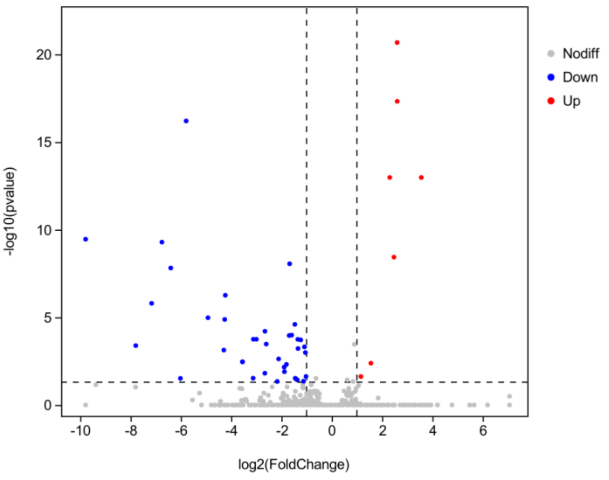

Figure S3. Gene expression profiles of *D. magna* after 9 d exposure to DES at 2 μg L^{-1} volcano plot compared with solvent control groups.

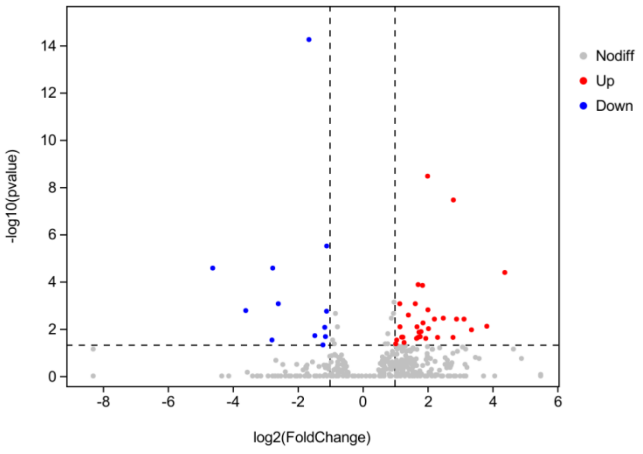

Figure S4. Gene expression profiles of *D. magna* after 9 d exposure to DES at 200 μg L^{-1} volcano plot compared with solvent control groups.

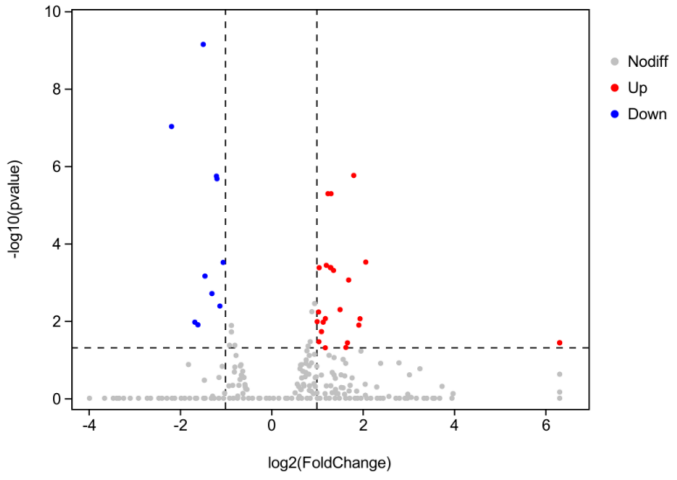

Figure S5. Gene expression profiles of *D. magna* after 9 d exposure to DES at 1000 μg L^{-1} volcano plot compared with solvent control groups.

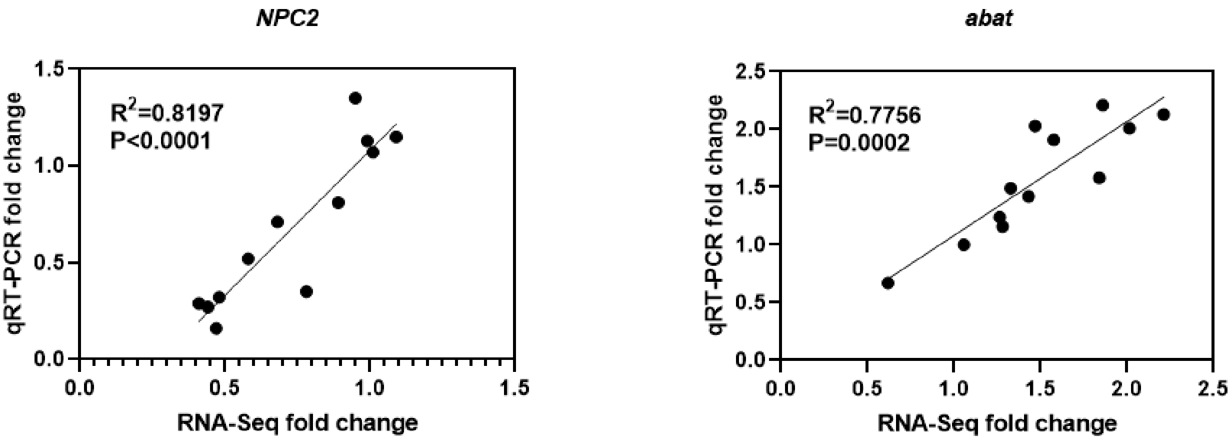

Figure S6. qRT-PCR and RNA-seq correlation analysis results diagram of selected genes. *NPC2:* Niemann-Pick C2 protein; *abat:* 4-aminobutyrate aminotransferase.

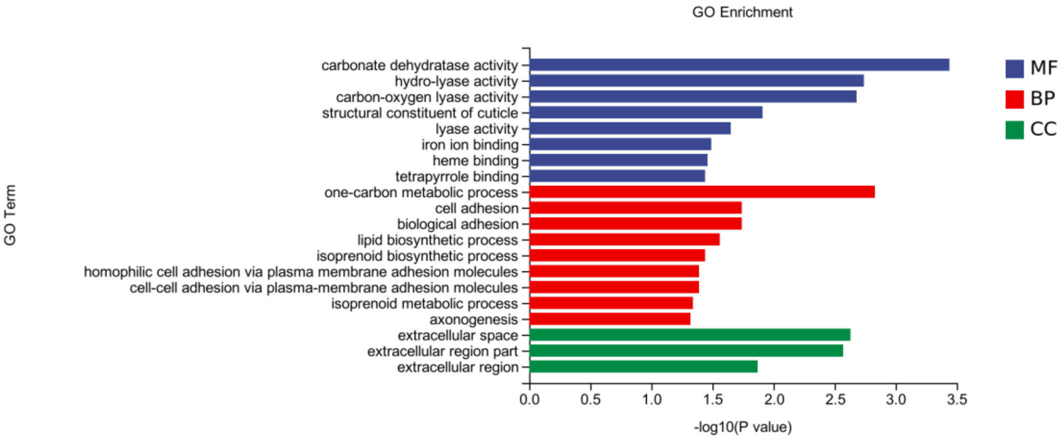

Figure S7. After a 9-d of exposure to different concentrations of DES, the top 20 enrichment terms for each category were significantly higher in the 2 μg L^{-1} treatment group than in the solvent control.

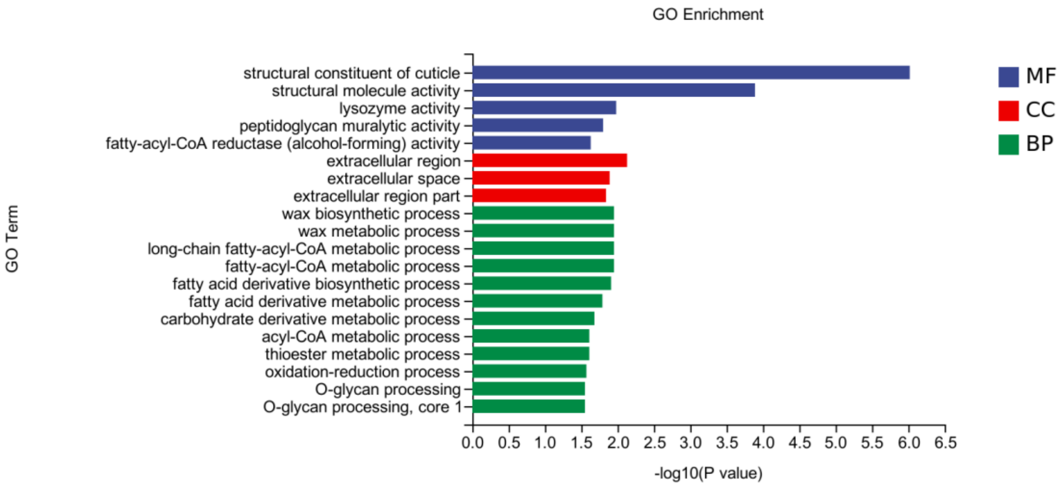

Figure S8. After a 9-d of exposure to different concentrations of DES, the top 20 enrichment terms for each category were significantly higher in the 200 μg L^{-1} treatment group than in the solvent control.

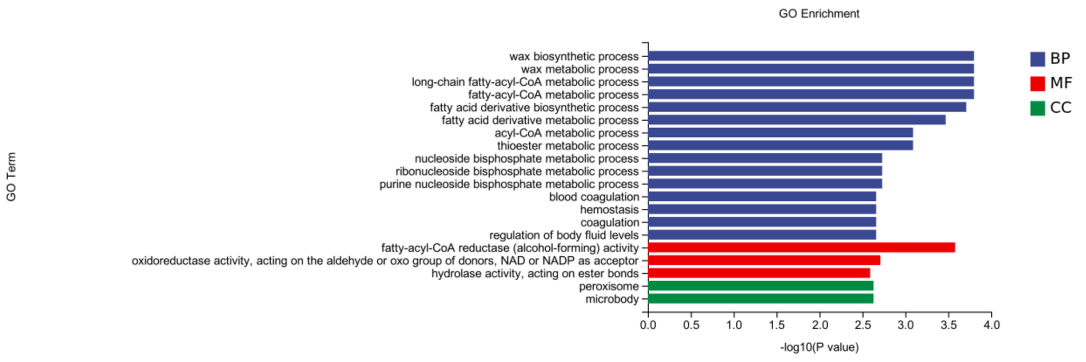

Figure S9. After a 9-d of exposure to different concentrations of DES, the top 20 enrichment terms for each category were significantly higher in the 200 μg L^{-1} treatment group than in the solvent control.

Table S2. List of fundamental pathways affected by a 9-day exposure to DES ($p < 0.05$).

Pathway ID	Pathway	Categary	Up-Gene	Down-Gene	*p*-Value	FDR
			**SC vs L**			
ko00910	Nitrogen metabolism	Energy metabolism	-	*ca*	0.34×10^{-3}	
ko00900	Terpenoid backbone biosynthesis	Metabolism of terpenoids and polyketides	-	*pdss*1	0.036	
ko00100	Steroid biosynthesis	Lipid metabolism	-	*meso*1, *erg*25	0.041	
ko04612	Antigen processing and presentation	Immune system	-	*ctsb*	0.042	
			**SC vs M**			
ko00073	Cutin, suberine and wax biosynthesis	Lipid metabolism	*far*	-	0.012	
ko04142	Lysosome	Transport and catabolism	*hgsnat*	*NPC*	0.014	
ko00531	Glycosaminoglycan degradation	Glycan biosynthesis and metabolism	*hgsnat*	-	0.016	
ko04979	Cholesterol metabolism	Digestive system	-	*NPC2*	0.043	
			**SC vs H**			
ko04979	Cholesterol metabolism	Digestive system	*LAL*	*NPC1, NPC2*	3.07×10^{-5}	
ko00073	Cutin, suberine and wax biosynthesis	Lipid metabolism	*far*	-	0.13×10^{-3}	
ko04142	Lysosome	Transport and catabolism	*lipa*	*NPC1, NPC2*	0.002	
ko04212	Longevity regulating pathway - worm	Aging	*far*	-	0.005	

ko04146	Peroxisome	Transport and catabolism	*far*	-	0.014
ko04974	Protein digestion and absorption	Digestive system	*prss, collagen*	-	0.018
ko00250	Alanine, aspartate and glutamate metabolism	Amino acid metabolism	*abat*	-	0.049
